# Testing Neuronal Accounts of Anisotropic Motion Perception with Computational Modelling

**DOI:** 10.1371/journal.pone.0113061

**Published:** 2014-11-19

**Authors:** William Wong, Nicholas Seow Chiang Price

**Affiliations:** Department of Physiology, Monash University, Victoria, Australia; Centre de Neuroscience Cognitive, France

## Abstract

There is an over-representation of neurons in early visual cortical areas that respond most strongly to cardinal (horizontal and vertical) orientations and directions of visual stimuli, and cardinal- and oblique-preferring neurons are reported to have different tuning curves. Collectively, these neuronal anisotropies can explain two commonly-reported phenomena of motion perception – the oblique effect and reference repulsion – but it remains unclear whether neuronal anisotropies can simultaneously account for both perceptual effects. We show in psychophysical experiments that reference repulsion and the oblique effect do not depend on the duration of a moving stimulus, and that brief adaptation to a single direction simultaneously causes a reference repulsion in the orientation domain, and the inverse of the oblique effect in the direction domain. We attempted to link these results to underlying neuronal anisotropies by implementing a large family of neuronal decoding models with parametrically varied levels of anisotropy in neuronal direction-tuning preferences, tuning bandwidths and spiking rates. Surprisingly, no model instantiation was able to satisfactorily explain our perceptual data. We argue that the oblique effect arises from the anisotropic distribution of preferred directions evident in V1 and MT, but that reference repulsion occurs separately, perhaps reflecting a process of categorisation occurring in higher-order cortical areas.

## Introduction

The sensory environment in which the brain finds itself is not altogether unpredictable. Certain categories of stimuli present themselves more frequently than others; for example, cardinal orientations and motion directions (horizontal and vertical) are common in natural visual scenes, and ubiquitous in artificial environments. Reflecting these environmental anisotropies, measurable biases in human perception and discrimination are also anisotropic, with most observers better able to detect and discriminate near-cardinal stimuli than oblique stimuli. Anisotropies are also evident in the stimulus preferences of sensory systems [1], with neurons that prefer horizontal and vertical orientations and directions the most prevalent in primary visual cortex [2], [3]. While the notion that anisotropic neuronal populations give rise to anisotropic perception is an attractive explanation for well-known perceptual biases, it remains unclear exactly how neuronal and perceptual anisotropies are linked. How can these similar environmental, neuronal and perceptual anisotropies be reconciled, and what can they tell us about how neuronal activity is both influenced by the environment and underlies perception?

We investigate two anisotropic phenomena that are prominent in motion perception: the oblique effect for motion, and reference repulsion [4]. Furthermore, we ask, do these perceptual effects originate from a single neuronal population, or might they depend on mutually exclusive populations? The oblique effect describes the observation that sensitivity to oblique motion directions is worse than that to cardinal directions [5], [6]. On the other hand, reference repulsion describes the somewhat controversial observation that judgements of motion directions tend to be repelled from certain “reference” directions, even though the reference may not be present in the stimulus [7], [8]. The cardinal directions are the most commonly reported reference directions; however, this finding is highly variable between study designs and individual subjects [9]. In particular, adaptation and other stimulus-driven priors can lead to repulsion from non-cardinal reference directions [10]–[15]. This flexibility in perceptual references suggests that neuronal anisotropies are not fixed, but can modified on timescales of a few seconds.

Although the oblique effect and reference repulsion have been simultaneously demonstrated in psychophysical experiments [4], to our knowledge, no studies have considered how a single anisotropic distribution of direction-selective neurons can account for both phenomena because they typically focus on just one perceptual effect. Most neuronal population decoding models of orientation and direction perception assume a population with uniformly distributed preferred orientations (or directions), tuning bandwidths, response amplitudes and read-out weights. Clearly, these rotationally-invariant models cannot explain anisotropies in perception. Anisotropic models have been created previously by either systematically varying tuning properties, or by incorporating prior (Bayesian) knowledge about the natural environment. Gilbert and Wiesel [17] systematically explored a range of context-induced anisotropies in orientation-tuned neurons, in order to explain the perceptual repulsion observed in the tilt aftereffect. Although they did not consider the oblique effect, they showed that adaptation-induced repulsion of orientation can be explained by three independent types of tuning changes: (1) a reduction in the gain of neurons that prefer orientations close to the adaptor; (2) a concentration in population tuning, so that more neurons prefer orientations close to the adaptor; (3) a narrowing of individual tuning, so that neurons that prefer orientations close to the adaptor have the smallest tuning bandwidths. Girshick et al. [1] developed a model to explain the oblique effect using a Bayesian estimator with priors based on orientation distributions in natural scenes. Like other models developed specifically to explain the oblique effect, this produced cardinal *attraction*, the opposite of what is commonly reported [1], [13], [17].

While previous modelling results have demonstrated that neural anisotropies can easily account for perceptual anisotropy, they have not attempted to simultaneously account for *both* reference repulsion and the oblique effect. Sensibly, if a single population *can* account for both effects, it should be given prime consideration by *lex parsimoniae*, and physiological studies have not suggested the existence of multiple, parallel populations of motion-sensitive neurons that contribute to perception [18]. In addition, the duration over which the population activity is decoded to generate perception has had little consideration in the past, and yet we demonstrate in this study that the duration over which modelled populations integrate their activity is probably as important for modelling results as the tuning characteristics of neurons themselves in some maximum likelihood decoding paradigms [19]. Facing these open issues, we quantified the timescales of the oblique effect and reference repulsion using an analogue-reporting technique. We further examined the interaction between these “innate”, or environmentally-driven, anisotropies and those introduced by brief adaptation to a single direction. To explain the perceptual anisotropies, we systematically explored vector averaging and maximum likelihood decoding models based on neuronal populations with anisotropies in their distribution of preferred directions, bandwidths and peak firing rates. We argue that the oblique effect arises from the previously reported anisotropic distribution of preferred directions evident in V1 and middle temporal area (MT), but that reference repulsion occurs separately, perhaps reflecting a process of categorisation occurring in higher-order cortical areas.

## Methods

### Participants

Twenty-two people with normal or corrected-to-normal visual acuity participated in the experiments – thirteen in Experiment 1 and twelve in Experiment 2, with three in common. Three experienced psychophysical observers (including the authors) participated in Experiment 1. The remaining participants were volunteers who received financial compensation for their participation and were naïve as to the procedures of the experiments. Experiments were approved by the Monash University Human Research Ethics Committee, and all participants gave informed written consent.

### Stimulus and Task

Stimuli were generated using Matlab (The Mathworks, Natick, MA) and the Psychophysics Toolbox extensions [20], [21]. Stimuli were presented on a Sony Multiscan G500 CRT monitor (40 cm width, 100 Hz refresh rate, 1024×768 pixel resolution) connected to a desktop computer under the Windows XP Professional operating system with an ATI Radeon HD 3400 Series graphics card.

We used random-dot motion stimuli comprising black dots (10.6 cd/m^2^) on a white background (115 cd/m^2^) displayed at 100 frames per second. Each dot was an anti-aliased circle of 4 pixel radius and displayed within an invisible aperture centred on the screen with a diameter that subtends a 15 deg viewing angle. (Note that we use “deg” to indicate spatial angles subtended at the eye (e.g. for speeds and stimulus size) and “°” to indicate rotational angles on the screen (e.g. motion directions).) In the first frame of the stimulus, dots were randomly plotted within the aperture at a density of 3 dots/deg^2^. For each successive frame, a random 90% subset of the dots moved in the same direction with a speed of 5 deg/s. The remaining 10% of dots were randomly replotted within the 15 deg aperture, giving a median dot lifetime of 66 ms. Any dots that moved outside the aperture were replotted in a position reflected about the aperture centre, maintaining uniform dot density across time. The 90% signal coherence prevented participants from tracking the local motion of individual dots or clusters of dots.

All stimuli were viewed binocularly from a distance of 70 cm, roughly giving a 30.8 px/deg grid resolution. A chin and forehead rest was used to stabilise the head and minimise eye movements. In addition, participants were specifically instructed to fixate a red 0.1 deg radius spot in the centre of the screen and to avoid tracking the motion of any individual dots. A 2 deg radius annulus surrounded the fixation spot, ensuring that no moving dots crossed the point of fixation, as a measure to discourage reflexive eye movements. We did not measure eye movements, but a previous eye-tracking study with similar methods did not observe significant eye movements provided the fixation spot and annulus were used [22].

#### Experiment 1: Anisotropy and adaptation effects on motion judgement

**Figure 1:**
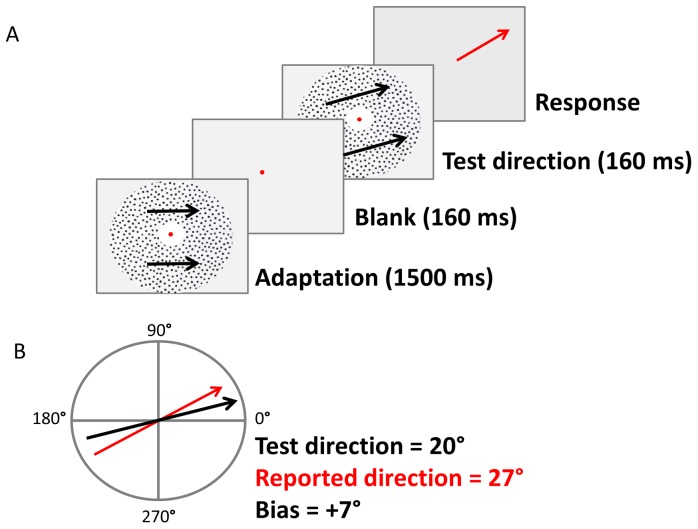
Trial sequence for Experiment 1. (A) Each trial began with the presentation of the experimental motion for 1500 ms, followed by 160 ms of a blank screen. A test stimulus with a randomly chosen direction was then presented for 160 ms, followed by a blank screen. The participant reported their perceived direction of the test motion by extruding a red response arrow from the centre of the screen using a computer mouse. The error (bias) on each trial was calculated as the difference between the true and reported directions (B).

Experiment 1 quantified how the precision and accuracy of direction judgments depended on stimulus direction, and how perception was affected by adaptation. Each trial contained 4 periods: (1) a 1500 ms “adaptation” period with either static noise dots as a control (90% coherence but 0 deg/s speed, giving no net signal motion and a slow flicker), or moving dots as the adapting stimulus with speed 5 deg/s in direction 0° or 45°; (2) a 160 ms “blank” period; (3) a 160 ms “test” period containing a signal motion; and (4) a “response” period (Figure 1). The fixed duration of the first two periods provided a visual timing cue for the appearance of the test stimulus. The signal direction during the “test” period was chosen randomly from a uniform circular distribution, rounded to the nearest multiple of 10°.

Although we used quantised test directions, it is unlikely that observers could identify when a particular test direction was presented. In pilot studies with just three adjacent test directions, observers could not reliably identify the test direction. For each observer, the number of trials in which the reported direction was within ±2.5° of any test direction was not significantly greater than the number of trials in which the reported direction was >2.5° from a test direction (one-tailed Wilcoxon rank sum test, U_n = 180+180_≥32162, p>0.05).

Participants indicated their perceived direction of the test motion during the “response” period by extruding a red arrow from the centre of the screen using a mouse. The arrow direction was recorded to the nearest degree. Similar methods of analogue report have been used previously and the presence of the oriented line does not appear to bias results [4], [23]. Participants had 5 seconds to initiate their response by pressing the left mouse button, and up to 10 seconds in total to finalise their responses by releasing the button. Any trials that “timed out” were not recorded. No feedback was given regarding response correctness.

Participants undertook from one to three experimental sessions that each lasted up to one hour. The first session included 5 minutes of practice trials to familiarise participants with the stimulus and procedure. The three adaptation conditions were presented in randomly ordered blocks of at least 50 trials of a single condition, and trials were presented in sets of 10, between which participants were allowed to rest at their own determination. In total, 15,908 trials were recorded, with each participant completing 220–1170 trials for each condition. We did not observe any systematic differences between participants who completed a small or large number of trials, and the variability in number of trials is not expected to bias the group results.

#### Experiment 2: Effects of stimulus duration on motion judgement

Experiment 2 quantified how stimulus duration affected the anisotropies evident in direction perception. Only the stimulus timing differed from the methods used in Experiment 1. First, the 1500 ms adaptation period was replaced with a 500 ms cueing period with static noise dots (90% coherence, 0 deg/s). Second, the duration of the test stimulus was varied in 5 conditions (20, 30, 40, 60 and 640 ms). A pilot test involving the three experienced participants had 6 duration conditions (20, 40, 80, 160, 320 and 640 ms). Blocks of 50 trials with each test duration were presented in random order and each participant completed 2–10 blocks of each condition (100–700 trials). In the pilot test, a total of 8,520 trials was recorded; and in the proper experiment, a total of 13,785 trials was recorded.

### Analysis

To evaluate each participant's performance, we initially calculated a “direction error” for each trial as the signed difference between the participant's response and the test direction. Our initial analyses indicated that when stimuli were of extremely short duration (<60 ms), many participants could reliably identify the orientation axis of motion, but not the true direction of motion. To simplify comparisons between stimuli with different durations, we primarily assessed “orientation errors”, focusing on deviations from the axis of stimulus motion. Thus, direction errors of 5° and 185° are both equivalent to an orientation error of 5°. Orientation errors are limited to the range ±90° relative to the axis of the test motion direction.

We assessed perceptual performance using four metrics, which were applied to trials with the same test direction, with the possibility of averaging across a group of test directions:

1. The “orientation bias” is the signed, circular median of the orientation errors
2. The “orientation accuracy” is the median of the absolute values of orientation biases
3. The “orientation precision” is the circular standard deviation of orientation errors
4. The “reversal fraction” is the proportion of trials in which the absolute direction error was greater than 90°.

In order to compare performance anisotropies across different stimulus durations, we defined three further metrics:

1. The “relative orientation accuracy” is the ratio of orientation accuracies at non-cardinal directions relative to cardinal directions
2. The “relative orientation precision” is the ratio of orientation precisions at non-cardinal directions relative to cardinal directions
3. The “reversal fraction difference” is the difference between arcsine-transformed reversal fractions at non-cardinal directions from cardinal directions.

### Population modelling

**Figure 2:**
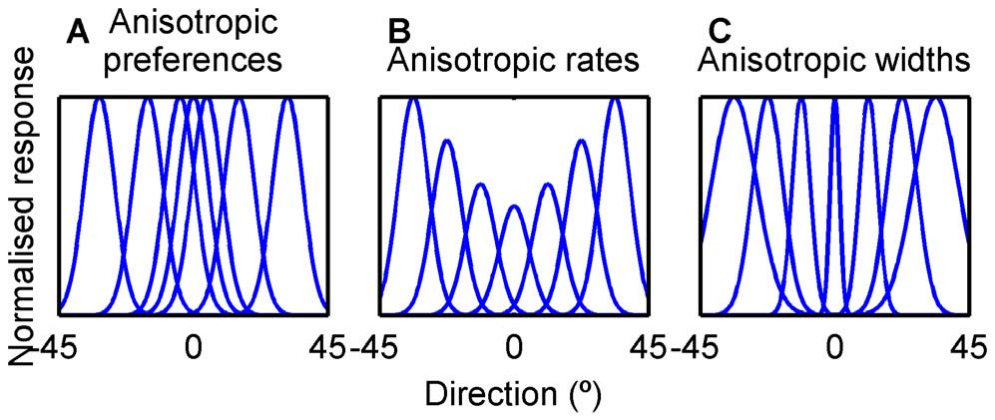
Population anisotropies. Three types of anisotropy that can be parametrically varied in the model; each curve represents the direction tuning of a single neuron. The model could independently specify anisotropies in the preferred direction (A), gain or peak firing rate (B), or tuning bandwidth (C). Note that for clarity, only one parameter is varied in each panel, but in the model, all parameters could be independently and simultaneously varied.

We broadly explored how anisotropies in the tuning of a single population of modelled MT neurons might explain our human perceptual data in the absence of adaptation. Starting only with a population whose distribution of tuning characteristics was specified based on that of macaque MT cells, we systematically manipulated the anisotropy of the distributions of three classically defined parameters: preferred direction, peak spiking rate and tuning bandwidth (Figure 2). Specifically, we varied anisotropy in a circularly-symmetric manner that was biased towards or away from the cardinal directions. Populations of 300 direction-selective neurons were simulated, with the response of each neuron to a single presentation of a stimulus direction drawn from a Poisson distribution with a mean defined by the neuron's direction tuning function (a circularly-wrapped Gaussian).

The distribution of preferred directions (θ_PD_) was described by a triangular function constrained to be symmetric about the cardinal axes:
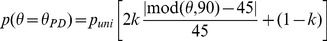
(Equation 1)


Where: *p_uni_* was the probability of each direction under assumptions of uniformity (1/36); and 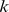 was varied in 7 steps between −1 and 1, producing distributions of *θ_PD_* that ranged from strongly favouring cardinal directions (k = 1, Figure 2A) to strongly favouring oblique directions (k = −1), and produced a uniform distribution when 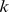 = 0. Direction tuning bandwidths and peak spiking rates for the modelled neurons were matched to previously reported properties of MT neurons [24], [25]. A pool of full-width at half-maximum direction tuning bandwidths was drawn from a piecewise linear distribution, with minimum 30°, maximum 160° and mode 70°. A second pool of peak spiking rates was drawn from a beta distribution with a range of 0–100 spikes/s, and shape parameters *α* = 1.2 and *β* = 5.

Our model allowed us to control the correlation between the cardinal distance of neuronal preferred directions and the anisotropic distributions of tuning bandwidths and response amplitudes. It was thus possible to systematically simulate different neuronal populations in which preferred directions, narrow tuning bandwidths and low response amplitudes are anisotropically distributed to either over-represent or under-represent cardinal directions. Each neuron was allocated a tuning width and spiking rate from the randomly generated pool based on the distance of its preferred direction from the nearest cardinal. While the parameter *k* in Equation 1 controls the level of anisotropy in preferred directions, two weighting factors control the level of anisotropy in peak spiking rate (w_R_) and tuning bandwidth (w_BW_). Both weights were limited to the range [−1 1]. For simplicity, positive levels of anisotropy correspond to those suggested by physiological literature [2], [3], [26], [27]: positive *k* concentrates neurons with preferred directions around the cardinals; positive *w_BW_* concentrates narrow bandwidths around the cardinals; and positive *w_R_* arbitrarily concentrates minimum peak firing rates around the cardinals.

**Figure 3:**
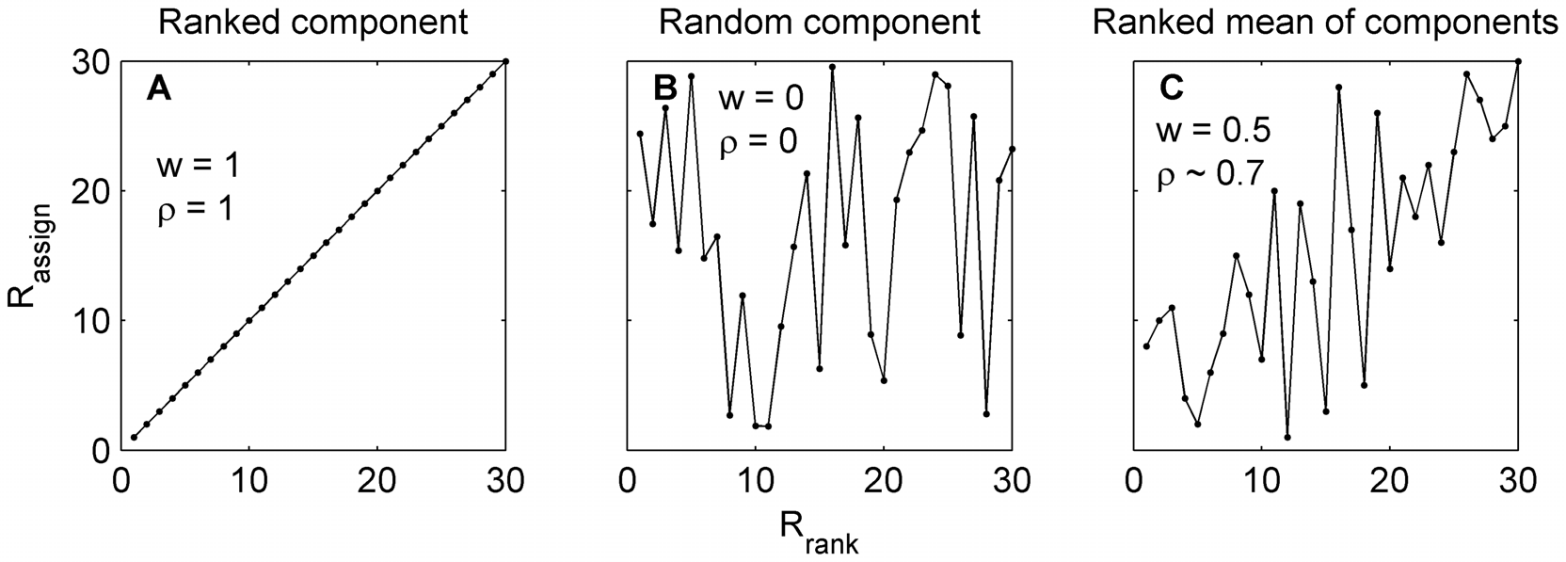
Assigning anisotropic tuning parameters based on a neuron's preferred direction. The tuning parameter value (bandwidth or peak spiking rate) given to each neuron in order of distance from cardinal may vary from being fully sorted (A) to entirely random (B). Intermediate levels of orderliness may be generated by a weighted average of the two extreme configurations by factor *w*; this is exemplified in (C) with w = 0.5, resulting in a rank assignment with Spearman rank correlation, ρ≈0.7.

Based on these weights, the degree of correlation between a neuron's preferred direction and tuning bandwidth or response amplitude could be easily and independently manipulated. The algorithm we formulated for generating the tuning assignments is:

1. Rank the neurons by the distance of their preferred direction from the nearest cardinal direction (*R_dir_*, Figure 3A);
2. Assign each neuron a random value in the range [1 n], where n is the number of neurons (*R_rand_*, Figure 3B);
3. Calculate a weighted sum of the two sets of ranks (*R_dir_* and *R_rand_*), 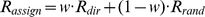 (Figure 3C); Note that this weighted sum was calculated independently for the weighting factors, *w_R_* and *w_BW_*
4. Sort the neurons by *R_assign_*;
5. Match the ordered pool of rate and width tuning parameter values to the order of the neurons, based on *R_assign_*.

To relate the resulting anisotropy to a statistical measurement, we have found that the average Spearman rank correlation of such generated sequences could be approximated as a function of the anisotropy weighting:
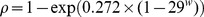
(Equation 2)


On average, *w* = 0 and 0.5 produces a number series with a rank correlation coefficient of 0 and 0.7, respectively (Figure 3). Weightings of *w* = 1 invariably result in a rank correlation coefficient of exactly 1.

We generated model populations of neurons in which parameters *k, w_BW_* and *w_R_* could take values from −1 to 1 in steps of ⅓, giving 7^3^ configurations of anisotropy. For each configuration, we also compared the results of four neuronal integration times, and tested two different methods of decoding the neuronal responses. Each configuration was instantiated 1000 times with randomly constructed neuronal tuning each time, resulting in 7^3^×4×2×1000 = 2,744,000 unique models. For each unique model, we predicted a perceived direction for five test directions (0, 10, 20, 30 & 40°). Note that due to the four-fold rotational and mirror symmetry of our model, these test directions are sufficient to quantify reference repulsion and the oblique effect for any test direction. Total simulation time was approximately one computer-month on two workstation PCs (processors Intel Core2 Duo E8600 and Intel Core i5-2400) under the Windows 7 Professional operating system.

The two neuronal decoding methods we tested were a vector averaging (VA) method and a maximum likelihood (ML) method. Our VA decoding method followed typical procedures [28], but additionally, vectors were weighted to equalise for peak spiking rate and total population activity at each neuron's preferred direction. Our ML decoding method extended that described by Jazayeri and Movshon [29], taking into account inhomogeneous populations and wrapped Gaussian tuning. For each decoder, we studied four neuronal integration times that produced orientation precisions similar to those found psychophysically: ML decoding used spike counts obtained over integration times of 1, 3, 5 & 25 ms; and VA decoding used 5, 20, 80 & 160 ms durations. This was simulated by drawing a spike count for each neuron, each from a Poisson distribution with lambda value the product of the expected spike rate and duration time. In pilot tests, we generated models with an even finer sampling of integration times, but report only a few for clarity. Based on the average predicted direction for each test direction, cardinal repulsion and oblique effect were quantified in a way that is analogous to the relative orientation accuracy and precision metrics for psychophysical data (Equations 3 and 4). Note that *μ_i_* and *σ_i_* are the bias and precision of the predicted perception for direction *i*, and we only considered the 5 test directions 0, 10, 20, 30 and 40°.
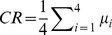
(Equation 3)

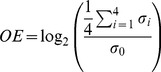
(Equation 4)


Matlab code that implements the model framework described here is provided as Supporting Information (File S1), including a demonstration script.

## Results

### Experiment 1: Perceptual anisotropy and adaptation

In Experiment 1, we assessed how adaptation affected bias, precision and reversal fraction of direction judgments. We used both cardinal and oblique adaptation directions allowing us to compare the interaction between anisotropies induced by recently viewed stimuli and those associated with “innate” perceptual anisotropies arising from directions that are over-represented in the environment.

**Figure 4:**
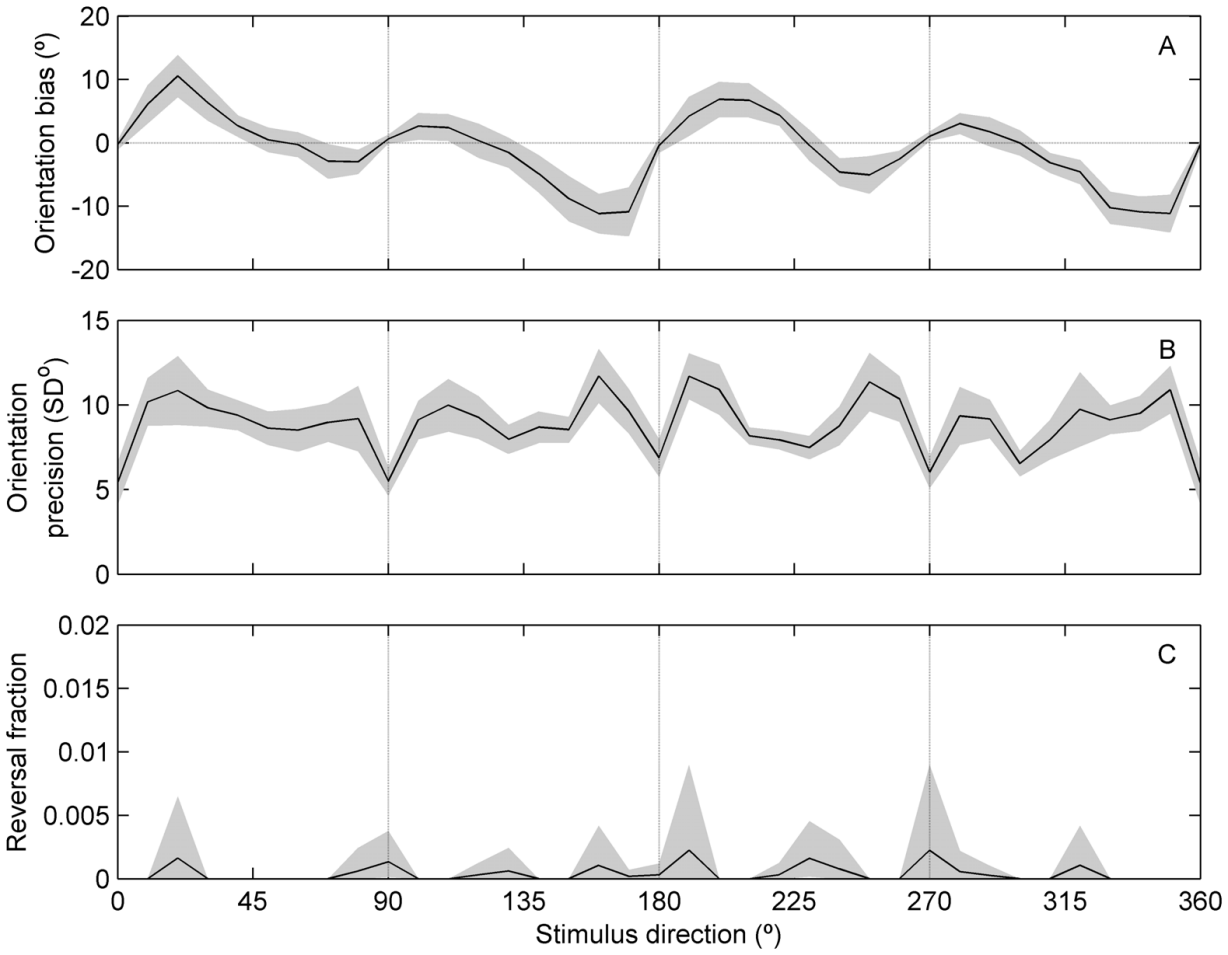
Direction-dependent perceptual performance. Bias, precision and reversal fraction have an anisotropic dependence on stimulus direction. All results show mean ± *SE* across 12 participants, based on the control data from Experiment 1 (stimulus duration of 160 ms). (A) Perceptual repulsion from horizontal directions is evident as the large positive slopes near 0 and 180°, and the smaller, but still positive slopes near 90 and 270°. (B) The oblique effect is apparent as the higher precision (standard deviation) for oblique directions compared to cardinal directions. (C) Reversals were negligible due to the long stimulus duration.

Both reference repulsion and the oblique effect for motion were evident in our results, with cardinal directions (0, 90, 180 and 270°) having the smallest biases and best precision (lowest standard deviation) in the control “no-adaptation” data (Figure 4). Due to the relatively long, 160 ms viewing duration, very few trials resulted in direction errors larger than ±90°, which we labelled as “reversals” (Figure 4C). Anisotropy was confirmed using mixed-design ANOVAs assessing orientation performances, grouped by stimulus direction and participant, with regards to bias (F_35, 12_ = 5.37, p<0.001) and precision (F_35, 12_ = 2.03, p = 0.001). Notably, differences between individual participants were also significant for orientation precision (F_12, 35_ = 10.8, p<0.001). In Experiment 2, significant within-participants differences were observed in both orientation precision (F_11, 35_ = 12.3, p<0.001) and bias (F_11, 35_ = 2.51, p = 0.004) for the 640 ms duration stimuli.

**Figure 5:**
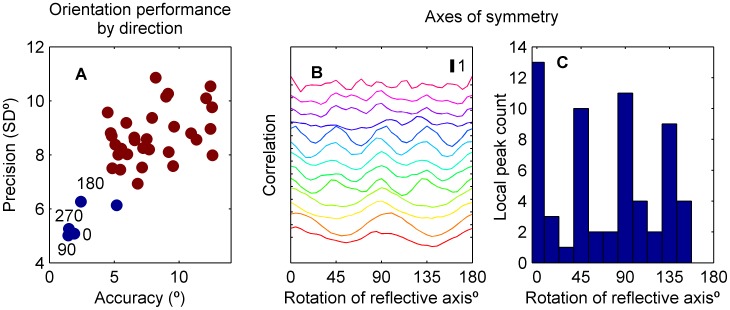
Analyses of cardinal-centric symmetry in orientation performance. (A) Comparison of population-mean precision and accuracy metrics illustrates that cardinal directions are associated with both higher accuracy and precision. Cluster analysis to the level of two groups resulted in one cluster containing the four cardinal directions and 300°. (B) The circular cross-correlation of each participants' direction-dependent orientation bias with its reflection – a measure of symmetry in the anisotropic judgement of directions. Each colour corresponds to a different participant, and curves have been vertically offset for clarity. Some participants exhibited correlations with 90° periodicity whereas others with 45° periodicity. In both cases, the highest correlations tended to occur across the cardinal axes. The histogram counts all local maxima in the cross-correlations of each participant; the most common axes of symmetry was indeed in the cardinal planes (0° and 90° rotation).

**Figure 6:**
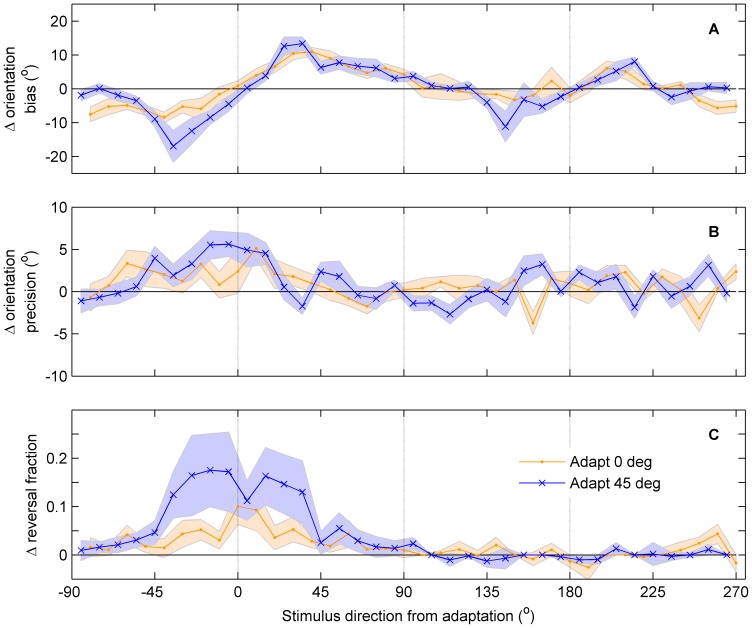
Adaptation. The change in performance of direction judgement following adaptation at 0° (red) and 45° (blue X), and their dependence on the stimulus direction relative to adaptation. Mean (± *SE*) changes in orientation accuracy (A), precision (B) and reversal fraction (C) are shown for 13 naïve participants. All values are expressed as *Adapt* – *Control*.

The special performance at cardinal directions was further demonstrated with a cluster analysis, using a standardised Euclidean metric and Ward's minimum variance method (Figure 5A). A distinct cluster of 5 directions (all cardinals plus 300°) was evident, suggesting that the cardinals are processed differently to oblique directions. To examine if the cardinal axes form lines of reflectional symmetry for the anisotropic effects, circular cross-correlations of orientation accuracy against its reflection were evaluated for reflection axes 0–180° in 5° steps, with linear interpolation of the underlying 10° interval data. Figure 5B shows these circular cross-correlations for each participant. We defined local correlation peaks as any axis for which the correlation value was higher than its immediate neighbours. Peaks in the circular cross-correlations correspond to the strongest axes of mirror symmetry. Across all participants, most peaks fall near multiples of 45° (Figure 6C). Notably, diagonal symmetry (peaks at 45 or 135°) was evident for the majority of participants, consistent with observers having similar accuracy for horizontal and vertical directions. The lack of this symmetry in some participants occurred because they exhibited only horizontal reference repulsion, evident in the population average (Figure 4A). Axes of symmetry were not evident in the circular cross-correlation of the orientation precision data.

We now examine how the “innate” perceptual anisotropies described above interact with those induced by adaptation to stimuli with directions of 0 and 45° (Figure 6). Under the assumption that the innate perceptual anisotropies reflect a long-timescale adaptation to environmental statistics, we predicted that adaptation to the cardinal 0° should compound the previously observed cardinal repulsion and oblique effect, whereas adaptation to 45° should introduce new biases, centred around 45°.

As we only tested observers with directions separated by 10°, our 45° adaptor falls in between two test directions. Therefore, the following group-wide analyses followed linear interpolation of individual participant performances over test directions in 5° steps. Adaptation induced perceptual repulsion (Figure 6A); across participants, the average orientation bias for directions 5–25° inclusively from the adaptation direction – our measure of repulsion – was significantly greater than 0 (average  = 5.7°; Wilcoxon signed rank test, W_12_ = 90, p<0.001). The strength of repulsion between 0° and 45° adaptation was not significantly different (W_12_ = 64, p = 0.210). Interestingly, the average repulsion within 5–25° of the direction *opposite* adaptation (180 or 225°) was also significantly greater than 0 (average  = 2.5°; W_12_ = 80, p = 0.013), but was smaller than the repulsion associated with the adapting direction; again, there was no significant difference in repulsion between 0° and 45° adaptation (W_12_ = 57, p = 0.444). These results are reminiscent of the direction aftereffect, which depends on direction-selective, not just orientation-selective mechanisms [30].

Orientation precision was significantly reduced (i.e. performance was impaired) for directions within 20° of the adaptation direction (W_12_ = 91, p<0.001), with 45° adaptation producing a significantly larger drop in precision than 0° adaptation (W_12_ = 74, p = 0.046). These precision drops could not be explained by just an increase in guessing because the reversed responses were clustered around the direction opposite the true direction and had standard deviation of 28±2°, smaller than that of a uniform distribution (52°).

Adaptation had no observable effect on perceptual precision for directions opposite the adaptor (Figure 6B). Reversal fraction was significantly increased for directions within ±20° of the adaptation direction, reminiscent of the repulsion that accompanies the motion aftereffect (W_12_ = 78, p<0.001). The difference in reversals between 0° and 45° adaptation was not significant (W_12_ = 61, p = 0.088). Again, there was no observable change in reversal fraction in the direction opposite adaptation (Figure 6C).

In summary, adaptation produced repulsion around the *axis* of the adaptor, with these effects compounding existing anisotropies when cardinal adaptation was employed. Adaptation decreased precision specifically around the adapting *direction*, which is contrary to the oblique effect. Additionally, reversals were affected only around the direction of adaptation.

### Experiment 2: Stimulus duration affects perceptual performance, but not anisotropy

**Figure 7:**
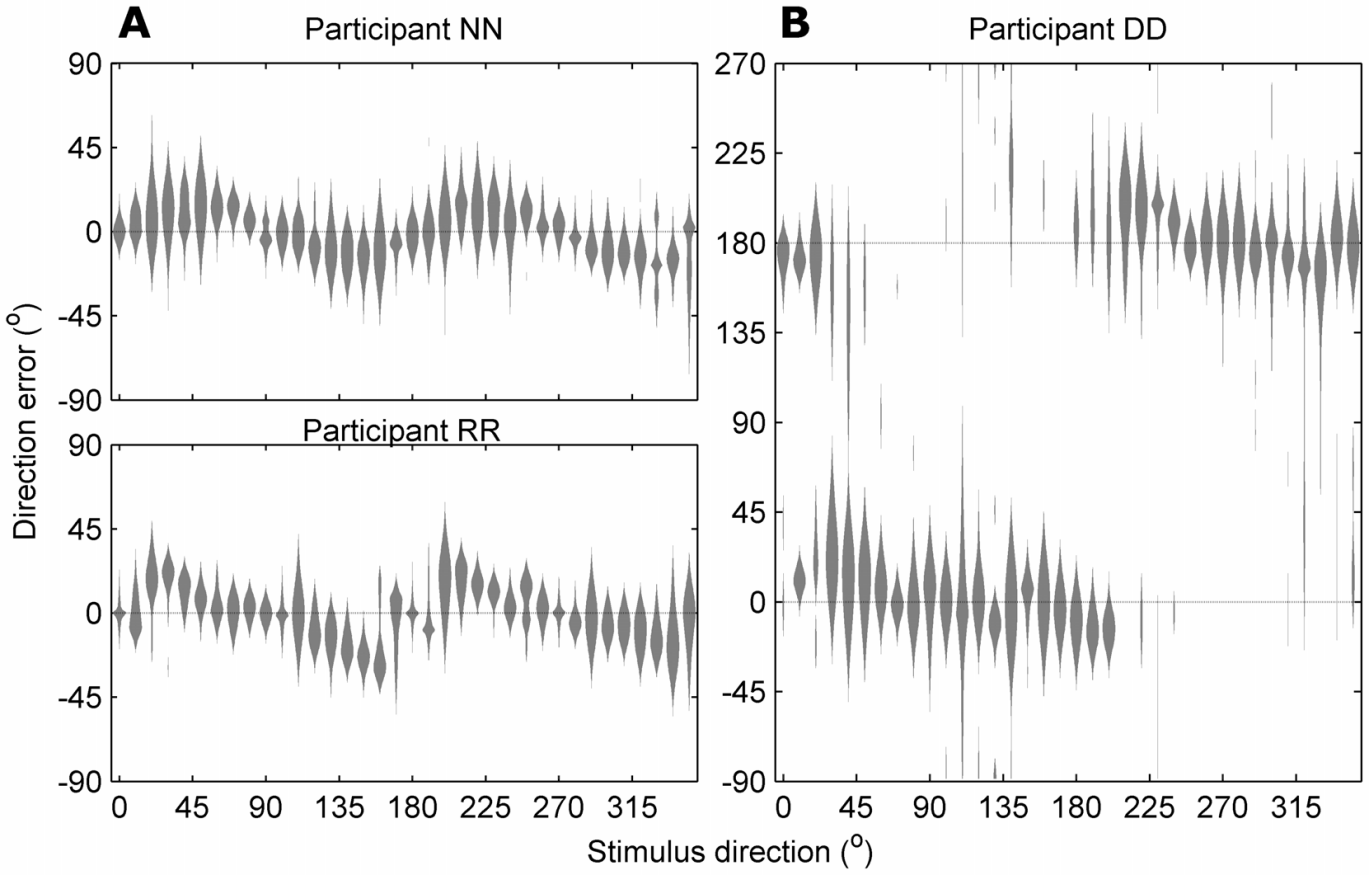
Individual performance. Distribution of errors in direction judgments, shown as violin plots for each stimulus direction (smoothed by adaptive-bandwidth kernel density estimation). Responses are shown for 3 observers from Experiment 2 trials with duration 160 ms (A) and 20 ms (B). Note the anisotropy in direction reports: cardinal directions tend to have more precise judgments, evident as a tighter distribution; near-cardinal directions tend to be reported as further from the cardinal axis than is true, leading to a bias in the mean error. At short viewing durations, many directions were reliably reported as 180° from the correct direction (B).

In Experiment 2, we examined how the duration of the motion stimulus affected performance. All participants had little difficulty judging motion direction when stimulus duration exceeded 80 ms. Figure 7A shows the distribution of errors for two observers for 160 ms stimuli. Errors are tightly clustered around 0° for all test directions, with 99% of errors not exceeding ±38°. In contrast, at short motion durations (20–80 ms), the distribution of perceptual errors tended to become bimodal with the emergence of “reversals”: a second cluster of errors near 180° (Figure 7B). The errors that exceeded ±90° had a significantly non-uniform distribution, suggesting that they were not due to random guessing (Rayleigh test for non-uniformity of circular data, p<0.001 for 10 participants; 2 participants were excluded because less than 50 reversals were recorded). Furthermore, we found that the mean orientation error from the *axis* of motion was not significantly different between unreversed and reversed errors in 9 of 10 participants (p>0.1). Thus, for simplicity, we classify all direction errors exceeding ±90° as “reversals”; and to allow comparison of motion perception between short and long duration trials, we quantified performance using the measures of orientation error and fraction of reversals.

**Figure 8:**
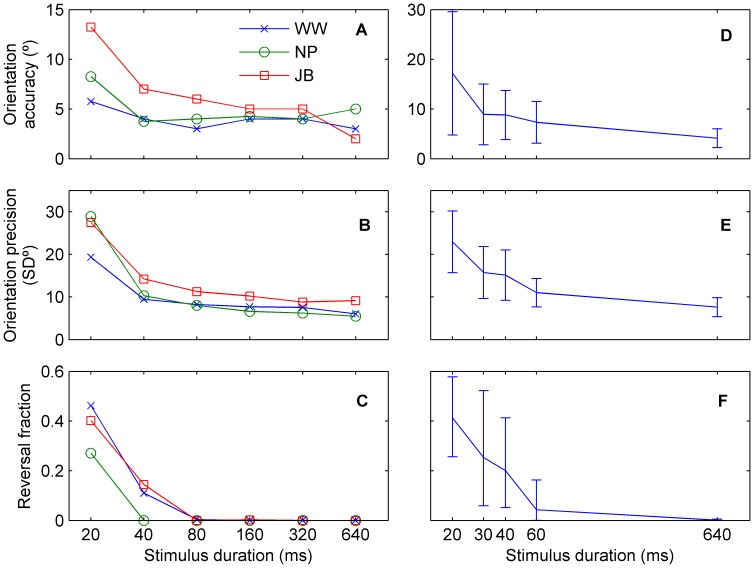
Duration-dependent perceptual performance. Increasing stimulus duration leads to better orientation accuracy (A, D), better orientation precision (B, E) and fewer reversals (C, F). Individual results are shown for three experienced observers (A–C) and for eleven participants (mean ± *SD*) tested over a different set of duration conditions (D–F).

Initial experiments with 3 experienced psychophysical observers demonstrated that longer stimulus durations led to improved accuracy and precision, and a reduction in the rate of reversals (Figure 8A–C). Impaired performance was primarily noticed for durations less than 80 ms; consequently, we more closely sampled short stimulus durations (20–60 ms) in 11 observers. The differences in performance between duration conditions were significant, as assessed by mixed-design ANOVAs, grouping stimulus duration and participant. Stimulus duration had a significant effect on all measures: orientation accuracy (F_4, 10_ = 10.6, p<0.001), orientation precision (F_4, 10_ = 35.3, p<0.001), and arcsine-transformed reversal fraction (F_4, 10_ = 44.4, p<0.001). Moreover, it was also clear that performance improved monotonically with increasing duration (Spearman's rank correlation: ρ_accuracy_ = −0.48, p<0.001; ρ_precision_ = −0.72, p<0.001; ρ_reversal_ = −0.78, p<0.001).

Even at the shortest stimulus duration of 20 ms (i.e. a single frame displacement of the stimulus dots), chance performance was not reached. For a large sample of trials with responses uniformly distributed in the range ±90°, accuracy and precision will approach 45° and ∼52°, respectively. However, to allow meaningful statistical testing, we defined probability distributions for chance-level accuracy, precision and reversal fraction separately for each participant using a resampling algorithm assuming errors distributed uniformly in the range ±90°. These probability distributions were computed over 5000 samples, assuming the same number of trials as actually tested for each participant, test direction and test duration. For all participants and for all stimulus durations of 30 ms or longer, orientation accuracy and precision were outside the 95% confidence interval of the resampled distributions. For the 20 ms duration, just 2 out of 11 participants had non-significant (p>0.05) orientation accuracy and 1 participant had non-significant orientation precision. However, for 6 out of 11 participants, the reversal fraction was not significantly different from chance.

At what stimulus duration does performance saturate? In order to estimate the integration time that gave 95% performance saturation, we fit modified Weibull cumulative distribution functions to each participant's data. In Equation 5, *y(t)* models the performance (accuracy, precision or reversals) at integration time *t*. Constants *a* and *b* control the lower and upper bounds, respectively, while *λ* and *c* are shaping parameters. For fitting accuracy and precision, we fixed b = 45° and 52°, respectively; for fitting reversals, we fixed *a* = 0 and *b* = 0.5. Equation 5 was fit to 100 bootstrap samples of the participant's raw data for each stimulus duration. The median R^2^ value of all fits was 0.88.
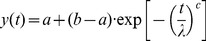
(Equation 5)


The median integration time for 95% maximum orientation accuracy was 26 ms with range 17–175 ms (n = 11), or range 24–91 ms (n = 7) when participants with fits with R^2^<0.5 were excluded due to minimally varying orientation accuracy. The geometric mean integration time for 95% maximum orientation precision was 56 ms (range 28.1–128 ms) and the geometric mean integration time for 95% maximum direction performance as measured by reversal fraction was 48 ms (range 22.7–80.2 ms).

**Figure 9:**
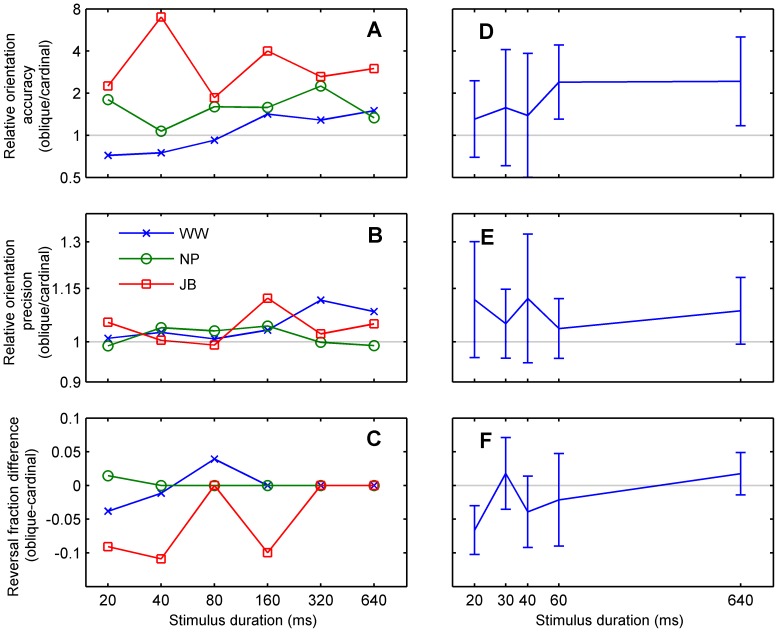
Relative performance for oblique versus cardinal directions. As in Figure 8, results are shown separately for three experienced observers (A–C) and averaged across eleven participants (D–F; mean ± *SD*). The ratio of oblique to cardinal orientation accuracy (A, D) and precision (B, E) was used to measure the strength of the oblique effect. Values greater than 1 indicate that orientation performance was better for cardinal directions than obliques. Error bars show geometric standard deviation. (C, F) The difference between arcsine-transformed oblique and cardinal reversal fractions; values greater than 0 indicate that direction performance was better for cardinal directions than obliques.

The perceptual anisotropies evident in Experiment 1 did not depend on viewing duration. Figure 9 shows the relative accuracy, precision and reversal fraction as a function of stimulus duration, expressed as the ratio of oblique and cardinal performance. Overall, there was no significant dependence of the relative orientation accuracy and precision on stimulus duration. Mixed-design ANOVAs assessing the log-transformed orientation anisotropy, grouped by stimulus duration and participant, showed no significant differences in relative orientation accuracy (F_4, 10_ = 2.24, p = 0.082) and relative orientation precision (F_4, 10_ = 0.94, p = 0.451). Reversal anisotropy, based on the arcsine-transformed difference between reversal fractions of cardinal and oblique directions, did significantly change with time (ANOVA, F_4, 10_ = 5.20, p = 0.002). A post-hoc analysis with Scheffé's method [31] showed reversals from 20 ms stimuli had a significantly lower reversal fraction difference than 30 ms stimuli (95% CI = [−0.157, −0.011]) and 640 ms stimuli (95% CI = [−0.157, −0.011]), suggesting relatively more cardinal reversals at the shorter duration. There were significant monotonic trends in relative orientation accuracy and reversal fraction difference by duration, but not for precision (Spearman's rho rank correlation; orientation accuracy anisotropy with log transformation: ρ = 0.311, p = 0.021; orientation precision anisotropy with log transformation: ρ = −0.039, p = 0.778; reversal fraction difference with arcsine transformation: ρ = 0.302, p = 0.025).

### Repulsion and oblique effect are observed in computational modelling of anisotropic MT neuron populations

We were interested in characterising what types of *neuronal* anisotropies could account for the observed *perceptual* anisotropies, and if the predicted level of neuronal anisotropy matches previously reported physiological data. We simulated a population of MT neurons in which we could systematically and independently vary the amount of anisotropy in the distributions of preferred directions, direction tuning bandwidths and peak firing rates. Decoding of this population activity was performed using both maximum-likelihood and vector averaging techniques. The resulting dataset is made available in Supporting Information (Dataset S1). To directly compare the model performance with our human data, we quantified the orientation precision for each model, as well as the strength of the oblique effect and cardinal repulsion in the model outputs (Equation 3 and 4).

For simplicity, we will first consider a single integration time for each model, chosen so that the orientation precision of an isotropic population was close to the ∼9° standard deviation observed for long viewing durations in our psychophysical data (Figure 8E). These integration times were 5 ms for ML decoding and 20 ms for VA decoding. To examine how each form of anisotropy contributed to cardinal repulsion and the oblique effect, we calculated the partial correlation between each type of neuronal anisotropy, and the strength of cardinal repulsion and oblique effect (Table 1). All partial correlations were large and highly significantly different from 0 (two-tailed t-test, absolute t_339_≥6.90, p<0.001), demonstrating that independently varying the distribution of neuronal preferred directions, firing rates or bandwidths can change perceptual anisotropy. As positive anisotropy metrics in the model (k, w_R_ and w_BW_) correspond with known neuronal anisotropies, we were surprised that the signs of the partial correlations were not always positive. Below, we primarily focus on the *sign* of the correlation, and whether changing the level of each type of neuronal anisotropy produces changes in cardinal repulsion and oblique effect consistent with perceptual data.

**Table 1:** Population anisotropy and decoding method on perception.

	Maximum likelihood		Vector averaging	
	Cardinal repulsion	Oblique effect	Cardinal repulsion	Oblique effect
Preferred direction (k)	−0.84	−0.62	−0.98	0.91
Spiking rate (w_R_)	0.76	0.70	0.35	0.53
Bandwidth (w_BW_)	−0.75	−0.80	−0.94	0.74

Spearman partial correlations between the strengths of two perceptual phenomena (the oblique effect and cardinal repulsion) and the level of anisotropy in preferred direction, spiking rate and bandwidth for integration times of 5 ms for ML, and 20 ms for VA. All correlations were significantly different from 0 (p<0.001).

For ML decoding, the cardinal repulsion and oblique effects consistent with our perceptual data were driven by neuronal anisotropies of fewer cardinal-preferring neurons, with cardinal-preferring neurons having the highest spike rates and broadest tuning bandwidths. Critically, each anisotropy produced the same sign of change in both cardinal repulsion and oblique effect (i.e. the partial correlations were both negative. In contrast, for VA decoding, more cardinal preferring neurons and narrower cardinal tuning bandwidths drives the oblique effect but also counteracts cardinal repulsion. This suggests that VA decoding cannot easily account for both the oblique effect and cardinal repulsion, because small changes in neuronal anisotropy will lead to changes in the two perceptual effects with the opposite sign.

**Figure 10:**
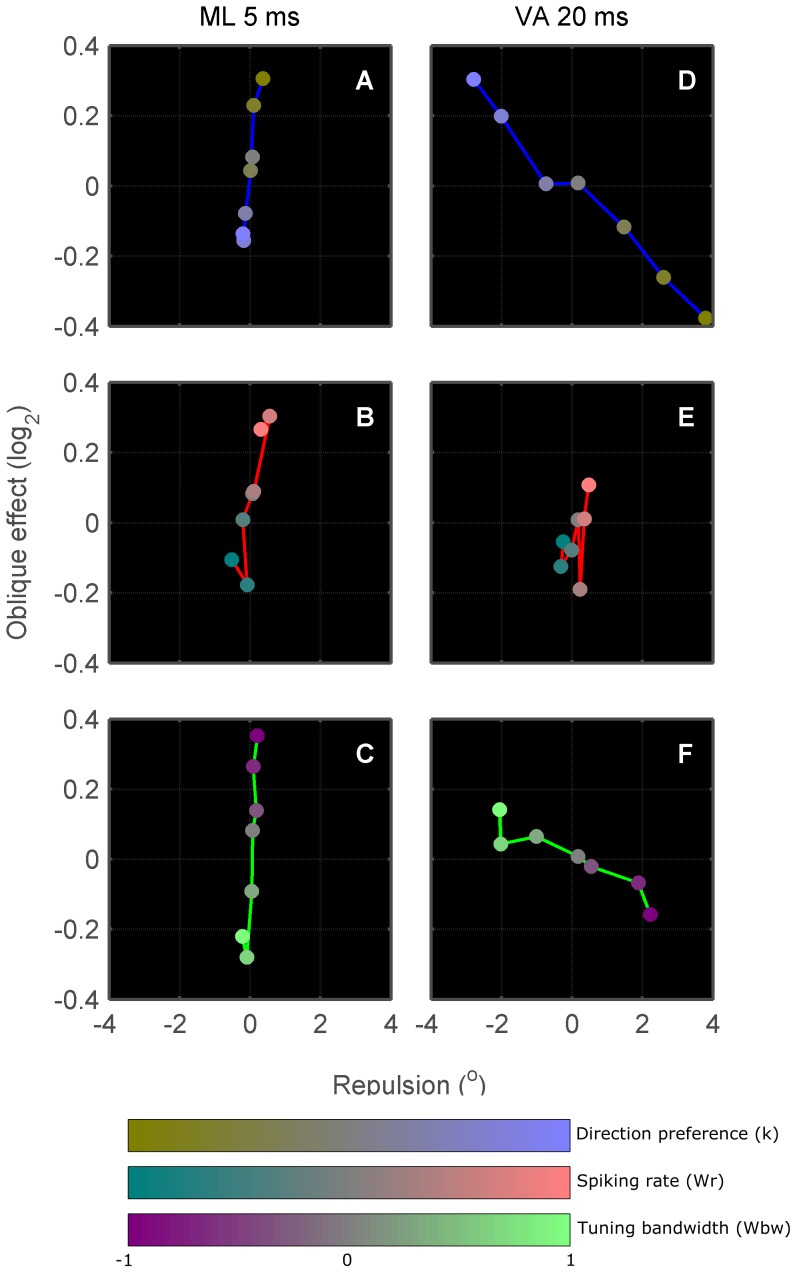
Isolated effects of population anisotropy. Maximum likelihood (A–C) and vector averaging (D–F) decoding of simulated anisotropic neuronal populations at integration times that produce similar perceptual precision to humans. Each coloured point quantifies the mean cardinal repulsion (Eq. 3) and oblique effect (Eq. 4) observed in 1000 instantiations of a neuronal population with a defined level of anisotropy. The colour of the point defines the level of anisotropy: mid-level (grey) RGB values correspond to neuronal populations with no anisotropies; higher blue values correspond to populations with more cardinal-preferring neurons; higher red values to lower spiking rates for neurons with cardinal preferences; and higher green values to narrower tuning curves for cardinal-preferring neurons. In each panel, the effect of varying a single anisotropy metric in isolation is shown: (A,D) variable anisotropy in direction preference (*k*) while enforcing no anisotropy in spiking rate or bandwidth (*w_R_* = 0; *w_BW_* = 0); (B,E) variable anisotropy in spiking rate (*w_R_*) while enforcing no anisotropy in direction preference or bandwidth (*k* = 0; *w_BW_* = 0); (C,F) variable anisotropy in bandwidth (*w_BW_*) while enforcing no anisotropy in direction preference or spiking rate (*k* = 0; *w_R_* = *0*).

**Figure 11:**
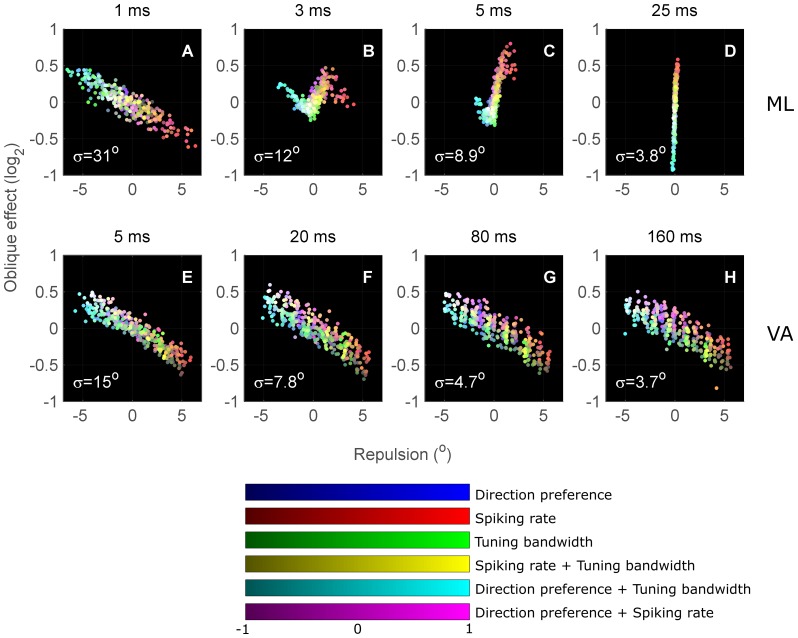
Combined effects of population anisotropy. Maximum likelihood (A–D) and vector average (E–H) decoding of simulated anisotropic neuronal populations. The colour mapping scheme is the same as in Figure 10. The first three colour bars map the intensities of each colour channel to a modelled anisotropy, and the last three colour bars demonstrate the additive effect of mixed anisotropies on colour. All the points in this figure express their anisotropy configuration as a sum of the three primary colours. Orientation precisions of the isotropic configuration in each panel are labelled with symbol *σ*.

Figure 10 illustrates the dependence of the oblique effect and cardinal repulsion on the level of anisotropy in preferred direction, spiking rate and bandwidth in isolation for both ML and VA decoding of neuronal activity. Each panel shows the performance of seven models corresponding to variations in only a single anisotropy metric while the other two anisotropy metrics are held constant. The level of neuronal anisotropy for each model is indicated by each data point's colour value, where each weighting variable corresponds to a primary colour component in the sRGB colour space (IEC 61966-2-1:1999) with linear spacing; *k* =  blue (preferred direction), *w_R_* =  red (spiking rate), *w_BW_* =  green (bandwidth). To reiterate: positive *k* concentrates neurons with preferred directions around the cardinals; positive *w_R_* concentrates minimum peak firing rates around the cardinals; and positive *w_BW_* concentrates narrow bandwidths around the cardinals. This modelling allows us to examine if there are specific forms of anisotropy that reproduce our perceptual data. Qualitative matches to our perceptual data required positive values for both the cardinal repulsion and oblique effect metrics, corresponding to data points falling in the upper-right quadrants of each panel. We further expect that as colour saturation increases, corresponding to increased strength of anisotropy, data points should move further into the upper-right quadrant. While this preliminary analysis indicates that a maximum likelihood decoder with anisotropy in spiking rate is the most realistic, this does not incorporate the possibility of a neural population with multiple anisotropies. Figure 11 addresses this by showing data for all combination of each anisotropy (243 models) and four different integration times. The average orientation precision produced by isotropic populations at each integration time is indicated in each panel.

A large family of ML decoders with red hue fall in the upper-right quadrant, suggesting that as long as anisotropies in peak spiking rate are present, neural decoding will match human perception and additional small changes in direction preference or tuning bandwidth will not strongly affect decoding. In contrast, for the VA decoder, points in the upper-right quadrant are relatively rare, primarily associated with red-magenta hues (spiking rate plus direction preference anisotropy). The orientation of the cloud of data points for the vector average decoder suggests that only specific combinations of anisotropy in direction preference and spiking rate can produce the expected perceptual effects. Thus, such a decoder would be relatively unstable in the face of small changes in neuronal anisotropy (as may be caused by adaptation), and is unlikely to underlie perception.

Drawing from known physiological anisotropies of primate visual area populations, the most realistic model configuration had *k* = ⅓, *w_R_* = 0, and *w_BW_* = ⅓, corresponding to a small over-representation of cardinal-preferring neurons, with those cardinal-preferring neurons tending to have narrower tuning bandwidths (see Discussion). The predicted perceptual results, using the aforementioned integration times that gave precisions most similar to the psychophysical data, was: repulsion  = −0.02°, oblique effect  = 2^−0.21^ for ML; and repulsion  = −1.9°, oblique effect  = 2^0.10^ for VA. With the realistic neuronal anisotropy, the only perceptual effect that could be qualitatively replicated (i.e. had positive sign) was the oblique effect under VA decoding.

We explored how the predicted perceptual effects depend on the modelled neuronal integration time (Figure 11). As integration time increases, the major change within the model is that the number of spikes available for decoding increases. While the shape of the cloud of VA decoders was robust across integration times of 5–160 ms, ML decoding showed large, complex changes in its behaviour. Notably, cardinal repulsion in the ML decoders is reduced at longer integration times. At the shortest duration of 1 ms, the effect of neuronal anisotropy on the oblique effect reverses, giving Spearman partial correlations of opposite sign to those in Table 1. It is interesting to note that for all model configurations (each unique colour in Figure 11 and its associated decoding method), integration time always has a significant effect on the amount of perceptual anisotropy; that is, there is a significant difference in measures of cardinal repulsion or oblique effect between at least 2 integration times for every configuration of neuronal population/decoding. All were tested by single-factor ANOVA, with each group by integration time containing 1000 bootstrapped measurements; p-values were adjusted for a Benjamini-Hochberg-Yekutieli false discovery rate [32] of 5% (ANOVA, F_3, 3996_≤17.4, p_adjusted_<0.001).

## Discussion

We examined how stimulus duration and adaptation affect the perception of motion direction in the fronto-parallel plane using an analog report method that was free of priming cues [23], [33]. Consistent with previous reports using two-alternative forced choice (2AFC) and analog reporting, we observed both reference repulsion and the oblique effect [4]. Our paradigm also permitted us to observe direction reversals and to demonstrate in a single experiment that some adaptation effects occurred in the orientation domain, whereas others occurred in the direction domain. This split is compatible with the notion of “motion streaks” at V1 contributing to direction perception [34], which provides a plausible explanation for the existence of reversals.

Reference repulsion was evident following adaptation, both in the direction matching and opposing the adaptor. For example, adaptation to 45° led to repulsion from both 45° and 225° directions, whilst adaptation to 0° added to the repulsion already associated with the cardinal axes. Our data is equivalent to an increase in the slope of the psychometric curve for a 2AFC task involving fine discrimination judgments of directions near the adaptor, which is consistent with previous reports that adaptation improves direction discrimination both near the adaptor and in the opposite direction [35], [36]. Surprisingly, adaptation increased the probability of reversals, and impaired precision, for near-adaptation test directions, but did not affect precision of directions opposite the adaptor. This is consistent with previous reports that adaptation impairs motion detection and noise-tolerance specifically near the adaptor [37]–[39], and the reversals are similar to the motion aftereffect [40]. Importantly, adaptation did not simultaneously enhance both reference repulsion and the oblique effect.

While longer viewing durations improved the accuracy and precision of direction perception, they did not systematically change the strength of cardinal repulsion and the oblique effect. This suggests that these perceptual anisotropies do not arise due to selective filtering of stimulus information over the duration of a single test stimulus (<160 ms), but reflect neuronal anisotropies that can change only on timescales of hundreds of milliseconds or longer. The integration time of motion direction perception across our participants was ∼50 ms, as measured by performance saturation of reversal fraction or orientation precision. This is comparable to integration times associated with motion streaks (∼77 ms), supporting a notion that motion streaks may contribute to the judgment of direction in our task [16], [34]. If this was the case, then in the 20 ms duration condition – where orientation performance dominated direction performance – the orientation cue would have been provided by streaks having moved just 19% of a feature width.

### Reconciling model predictions and known neuronal anisotropies

The aims of our neuronal population modelling were to determine the *simplest* form of anisotropy that could account for our perceptual results, and conversely, to determine what perceptual phenomena would be expected given the known forms of neuronal anisotropy. Historically, the observation of anisotropic neural tuning for orientation and direction has been controversial. Studies in primary visual cortices showed strong preference for cardinal orientations among individual neurons in macaques [27], [41] and in optical imaging of cortical territory in ferrets [42], [43]. However, numerous studies have also failed, at the single neuron level, to find any evidence for similar anisotropies or have reported insignificant trends towards anisotropy (e.g. [44]–[46]). A recent study suggested that orientation anisotropies are absent in V1, but emerge in V2 [47]. The difference in results possibly reflects the fact that most studies pool data from neurons with foveal and peripheral receptive fields [48]–[50]; the macaque V1 anisotropy was shown to be significant among neurons with foveal receptive fields, not peripheral.

In humans, fMRI studies routinely show that cardinal and oblique orientations and motion directions evoke *different* levels of activity. Some studies report lower activation for obliques [51], [52], whereas others report greater activation [2] or a greater number of cardinal-preferring voxels [53]. A bias towards over-representation of radial orientations and motion directions in human V1–V3 is also evident [54]. In imaging studies, the over-representation of regions activated by cardinally-aligned stimuli is usually interpreted as arising from an over-representation of cardinal-preferring neurons; however, it could also reflect anisotropically distributed spiking rates or direction-tuning bandwidths.

For single neurons, cardinal-preferring simple cells have been reported to have the narrowest tuning, whereas cardinal-preferring complex cells have the broadest tuning [55], which conforms with ideal models of neuronal populations embodying Bayesian inference about environmental statistics [56]. The most definitive study in this domain assessed over 4000 neurons in the central 15° of the visual field of area 17 in anaesthetised cats [26]: without regard for eccentricity, they found an over-representation of cardinal preferring neurons in simple cells tuned to orientations of high spatial frequencies, and these cardinal-preferring simple cells had the narrowest tuning bandwidths.

While no systematic anisotropies have yet been reported in single MT neurons – for which area is nominally associated with motion perception more than V1 (e.g. [18]) – optical imaging showed more cortical territory was devoted to cardinal directions in owl monkey MT, and this anisotropy is most prominent in the central 10° of vision [3]. The weight of studies strongly suggests that neural anisotropies are found in a range of species and early cortical areas, but they do not resolve what types of anisotropy dominate, and how they can account for the commonly reported perceptual anisotropies.

#### Modelled anisotropies connot simultaneously explain the oblique effect and reference repulsion

For our modelling investigation, we ignored the effects of adaptation and aimed to only model reference repulsion and the motion oblique effect at sufficiently long timescales for which direction reversals rarely occurred. The most important and surprising finding of our modelling was that known neuronal anisotropies in direction preference and tuning bandwidth (cyan points in Figure 11) did not result in both the oblique effect and reference repulsion, regardless of the decoding scenario or integration time. Rather than attempting to predict the most likely form of neural anisotropy by matching model outputs with our perceptual data, below we consider which models are least likely to be relevant.

First, we rejected maximum likelihood decoding because it showed an unstable dependence on the simulated neuronal integration time. Surprisingly, only the shortest integration time reproduced an earlier finding that anisotropic direction preferences and bandwidths produce the oblique effect [15]. Our model neurons had peak firing rates of up to 100 spikes/s, meaning that they likely fire just 0 or 1 spikes in a simulated trial lasting <10 ms. This suggests that the behaviour of the maximum-likelihood decoder changes as it enters a binary spiking regime, but the reason for this remains unclear. Although motion flow can be perceived in 2–4 ms [57], the nature of perceptual anisotropies at durations below 20 ms has not been explored.

Second, for the family of vector averaging models, the strength of the oblique effect and cardinal repulsion had opposing dependencies on the strength of anisotropy in preferred direction and tuning bandwidth. This suggests that a single anisotropic population of neurons cannot produce both perceptual effects simultaneously or would be unstable if neural tuning changed slightly; it would be more likely that different neuronal populations account for the two perceptual effects.

Finally, under VA decoding, the model configurations mimicking known neuronal anisotropies produced the oblique effect but not reference repulsion. In all, none of the modelled anisotropies were able to adequately explain the simultaneous oblique effect and reference repulsion of direction perception found in human behaviour, despite allowing neuronal tuning parameters to vary across the full range of anisotropies and keeping overall distributions of parameter values true to known physiological distributions. Therefore, we propose that it is most likely that well-characterised neuronal anisotropies in orientation and direction tuning can explain only the oblique effect, and that the perceptual decision based on the population response can be modelled like a vector averaging function. Consequently, we propose that reference repulsion originates from a different neuronal population with contrasting anisotropies beyond V1 and MT.

Our modelling incorporated neuronal anisotropies in a single cortical area; might different anisotropies in multiple sensory areas account for different perceptual anisotropies? A two-area model of perception could be built in two distinct ways: in a hierarchical model, information in one area would be inherited from an earlier area; and in a parallel model, two possibly-independent areas could each influence a different aspect of perception. A parallel model does not seem likely as such independent pathways are not known to exist for motion perception; further, as outlined in the previous section, the neural anisotropies described in areas V1, V2 and MT are all similar and should produce the same perceptual effect. Rokem and Silver [15] described a hierarchical model: anisotropy was introduced in an area of direction-selective neurons (representing V1), and inherited by a second area of neurons (representing MT). Critically, they showed that anisotropy in the first area propagates to the second area, regardless of whether there is any explicitly anisotropic wiring or neural distribution in the second area. Thus, a single-stage model should be able to incorporate all neuronal anisotropies present in a hierarchical multi-stage model.

A further limitation of our model is that the responses of each neuron in our model were independent, even though the strength of neuronal correlations in V1 and MT depends on similarities in tuning preference [58], [59]. If there are more cardinal-preferring neurons, and neurons with similar preferences have stronger connections, then the correlation structure across a population of neurons must also be anisotropic, which may further affect perceptual performance [60].

We suggest that it is unlikely that anisotropies in two sequentially-connected sensory areas could account for both the oblique effect and cardinal repulsion. Rather, the motion oblique effect may be primarily determined by the anisotropic distribution of orientation preferences in V1, which are inherited and possibly enhanced by MT. Its neuronal anisotropies may consist of a combination of cardinal-preferring neurons tending to greater density, lower spiking rate and narrower tuning bandwidths. Reference repulsion may then arise from a higher-order process of categorisation, in which subjective reports are weighted according to the expected distribution of stimuli in the environment (e.g. [61]). Indeed, it has previously been argued that reference repulsion in the motion domain may not have a sensory origin, but arises at higher-order cognitive stages associated with categorisation [62]. If so, then the requisite category-selective neurons, including those implicated in categorising direction, exist in the lateral intraparietal area and prefrontal cortex [63], [64]. Essentially, if observers treat a reference direction (either a cardinal or adaptor) as a category boundary, then when they are confident that a near-boundary stimulus does not align with the boundary, the stimulus is reported erroneously close to the *centre* of the chosen category and further from the actual boundary. Thus reference repulsion may be an effect of recall, and may not explicitly depend on the anisotropic properties of environmental stimuli or neuronal tuning properties.

## Supporting Information

Dataset S1 Modelling data. Decoded perception to all instantiations of modelled populations and tested directions in comma-separated values (CSV) file format.
(ZIP)

File S1 Modelling framework code. Matlab code that implements the population modelling framework described in Methods, including a demonstration script.
(ZIP)
